# Effectiveness and cost-effectiveness of a multidisciplinary intervention programme to prevent new falls and functional decline among elderly persons at risk: design of a replicated randomised controlled trial [ISRCTN64716113]

**DOI:** 10.1186/1471-2458-5-6

**Published:** 2005-01-14

**Authors:** Marike RC Hendriks, Jolanda CM van Haastregt, Joseph PM Diederiks, Silvia MAA Evers, Harry FJM Crebolder, Jacques ThM van Eijk

**Affiliations:** 1 Department of Health Care Studies, section Medical Sociology, Faculty of Health Sciences, Maastricht University, The Netherlands; 2 Department of Health Organisation Policy and Economics, Maastricht University, The Netherlands; 3 Department of General Practice, Maastricht University, The Netherlands

## Abstract

**Background:**

Falls are common among community-dwelling elderly people and can have a considerable impact on quality of life and healthcare costs. People who have sustained a fall are at greater risk of falling again.

We replicated a British randomised controlled trial which demonstrated the effectiveness of a multidisciplinary intervention programme to prevent falls.

The objective is to describe the design of a replication study evaluating a multidisciplinary intervention programme on recurrent falls and functional decline among elderly persons at risk. The study consists of an effect evaluation, an economic evaluation and a process evaluation.

**Methods/design:**

The programme is aimed at community-dwelling elderly people aged 65 years or over who have visited an accident and emergency department (A&E department) or a general practitioners' cooperative (GP cooperative) because of a fall.

The design involves a two-group randomised controlled trial. Participants are followed for twelve months after baseline. The intervention programme consists of a detailed medical and occupational therapy assessment with referral to relevant services if indicated. People in the control group receive usual care.

The main outcome measures of the effect evaluation are number of falls and daily functioning. The economic evaluation will be performed from a societal perspective. A process evaluation will be carried out to evaluate the feasibility of the intervention programme.

## Background

### Publishing the design of a study

This article describes the design of a replication of a randomised controlled trial (RCT) evaluating the effectiveness and cost-effectiveness of a multidisciplinary intervention programme to prevent further falls among elderly people at risk.

Publishing the design and protocol of a study before results are available is important for several reasons. A published protocol allows easier comparison between what was originally intended and hypothesised and what was actually done [1], and it gives readers greater insight into the methodological quality of a study. Furthermore, it has often been recognised that negative or adverse outcomes are less likely to be published [1-3]. Publishing the design of a study before its start announces that a study will be undertaken, which encourages publication of the results and in any case informs researchers where they can find the data for inclusion in systematic reviews [1,2]. Thus, publishing a design article can prevent publication bias.

### Prevention of falls

About one-third of people over the age of 65 fall at least once a year [4]. People who have fallen show an increase in morbidity, mortality and healthcare utilisation [5], which implies increased healthcare costs. In addition, people who have sustained a fall are at greater risk of falling again [5].

Since preventing falls has been a matter of interest for years, many programmes aimed at preventing falls have been developed and evaluated. Unfortunately, many of these have turned out to be ineffective [4]. However, there is now considerable evidence of the effectiveness of multifaceted interventions. Programmes likely to be effective in preventing falls among elderly people are multidisciplinary, multifactorial programmes screening for health and environmental risk factors and offering interventions, both for the general population of community-dwelling elderly people and for elderly people with a history of falling selected because of known risk factors [4]. An example of such an intervention programme is the successful programme developed by Close et al. [5]. This programme is aimed at people aged 65 years or older who live in the community and have visited an accident and emergency department because of a fall. The intervention programme consists of a detailed medical and occupational therapy assessment with referral to relevant services if indicated. The intervention has been evaluated in a randomised controlled trial, which demonstrated that this multidisciplinary intervention implemented among people at risk was highly effective in reducing the number of recurrent falls and associated injuries in London (United Kingdom) [5].

Because details of the status of the participants, the context of the intervention and the content and presentation appear to be critical, it has been recommended to re-evaluate effective intervention programmes in different healthcare systems [4]. We therefore decided to evaluate the effectiveness of the intervention developed by Close [5] in Dutch healthcare, by replicating this study in the Netherlands.

### Objective and research questions

The main objective of our current study is to evaluate the effects of a multidisciplinary intervention programme on recurrent falls and functional decline among elderly persons who have visit a general practitioners' cooperative (GP cooperative) and/or an accident and emergency department (A&E department) because of a fall. This objective has resulted in the following research questions.

• Is a multidisciplinary intervention programme more effective than usual care in preventing new falls and functional decline among community-dwelling elderly people who visit a GP cooperative and/or A&E department at a hospital because of a fall?

• Is the multidisciplinary intervention programme cost-effective compared to usual care when assessed from a societal perspective?

Besides the effect evaluation and economic evaluation, a process evaluation is being carried out to assess the feasibility and applicability of the intervention programme for those receiving and implementing the intervention.

## Design and methods

### Design

**Figure 1:**
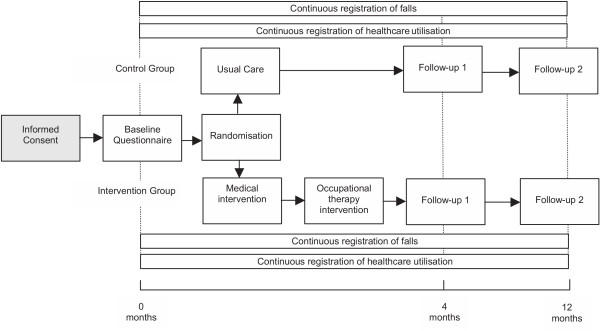
Study Design

Figure 1 shows the design of the study presented, which is a two-group randomised controlled trial. At this stage, the randomisation process has already been completed. Randomisation was achieved by means of computerised alternative allocation and performed by an external agency. Randomisation took place after completion of a self-administered baseline questionnaire. People allocated to the control group received usual healthcare, while people in the intervention group underwent a medical and occupational therapy assessment. The intervention period is scheduled to last for a maximum of 3.5 months after the baseline measurement. After baseline measurement, all subjects are followed for a twelve-month period. During this follow-up period, falls and healthcare utilisation are measured continuously. Subjects are contacted monthly by telephone for an interview about their falls and healthcare utilisation. In addition, self-administered questionnaires are sent to the subjects after four and twelve months.

We have taken various measures to ensure blinding in the data collection process. Questionnaires are collected anonymously and sorted by number. Follow-up measurements by phone are contracted out to an independent call centre, whose operators are unaware whether the subjects have been allocated to the intervention or the control group.

The study design and protocols were approved by the Medical Ethics Committee of the University Hospital and University of Maastricht.

### Target population

Various studies have been conducted to assess the effectiveness of programmes to prevent falls. Although most studies were aimed at the general population of elderly people, details of the status of the participants appear to be critical [4,6]. Several authors have suggested that interventions are likely to have greater effect when targeting people at risk [7,8]. People who attend an A&E department with an injurious fall form a high-risk group, and are expected to be more receptive to an intervention programme aimed at reducing falls than the general population of community-dwelling elderly people. In a study by Close et al., about half of the patients who attended an A&E department with a fall had experienced an earlier fall in the previous year, compared to about one third of the elderly people in the general population [5]. Like Close et al. [5], we chose community-dwelling elderly people aged 65 years or over who had sustained an injurious fall as the target population of our intervention programme. The following definition of a fall was used: 'A fall is an event which results in a person coming to rest inadvertently on the ground or other lower level'. This definition is derived from that used by the Kellogg International Work Group [9].

### Recruitment of the study population

Recruitment of subjects took place at the local GP cooperative and the A&E department of the University Hospital in Maastricht. The Maastricht GP cooperative is a group of GPs from practices in the town of Maastricht and the surrounding area who have founded a non-profit organisation to provide care for their own patients after hours [10]. The Maastricht GP cooperative has been set up at the hospital's A&E department and covers the out-of-hours service for all local GPs [11].

The following inclusion criteria were used: age 65 years or older, community-dwelling, having visited the A&E department or GP cooperative at the University Hospital Maastricht for the consequences of a fall, and living in Maastricht or its surrounding area. People were only allowed to enter the programme after completing and returning an informed consent form. Exclusion criteria were: not able to speak or understand Dutch, not able to complete questionnaires or interviews by telephone, cognitive impairment (a score of less than 4 on the Abbreviated Mental Test 4 (AMT 4) [12,13], long-term admission to a hospital or other institution (more than four weeks from the date of inclusion), permanently bedridden, or fully dependent on a wheelchair.

### Sample size calculation

Sample size calculations were based on the expected effects of the intervention on the main outcome measure, the percentage of people sustaining a fall during one year of follow-up. The study by Close et al. [5] found that the percentage of persons who sustained a recurrent fall was 52% in the usual care group and 32% in the intervention group. If we want to detect the same reduction in the percentage of persons sustaining a recurrent fall in our study, with a power (1-beta) of 90% and alpha of 0.05, we need 123 patients in each group (a total of 246). Based on the experiences of Close et al. and our own experiences in trials among elderly people in the Netherlands [14], we expect a dropout rate of about 25% during the one-year follow-up period. This means that about 164 persons per group (a total of 328) have to be included in the study. The inclusion period was 14 months.

### Intervention programme

To adapt the programme developed by Close et al. [5] to the Dutch situation, and to make improvements based on recent insights, we performed a review of the literature, convened a consensus meeting and tested the adapted version in a pilot study (n = 36). Based on this process, we made some improvements to Close et al.'s programme.

The final programme includes a medical and occupational therapy assessment resulting in recommendations. The medical assessment consists of an examination performed by a geriatrician, a geriatric nurse and a rehabilitation physician to identify and address risk factors for falling. The examination includes a comprehensive general examination, but in addition focussed on a more detailed assessment of visual acuity, stereoscopic vision, mobility, balance, cognition, affect, use of medication and examination of feet and footwear. Recommendations or indications for referral resulting from this examination are sent to the patient's GP. After the medical assessments, an occupational therapist visits the patients to identify possible risk factors for falling in the home environment. The therapist makes recommendations regarding adaptations to the home environment, assistive devices, home care and behavioural change. Recommendations by the occupational therapist are sent directly to the subjects themselves and to their GPs. As stated before, the intervention period is scheduled to last for a maximum of 3.5 months after the baseline measurement.

An important addition to Close et al.'s protocol [5] is the collaboration with a rehabilitation physician (physiatrist) in the medical part of the intervention. In addition to the screening by a geriatrician, our programme also involves screening by a rehabilitation physician who examines the subjects' feet and the shoes which the subjects wore at the time of the fall. Details of the process of adaptation and the contents of the intervention programme will be published elsewhere.

### Usual care in the Netherlands

People in the control group receive usual care. At present, no guidelines exist for the systematic assessment of the underlying causes of an injurious fall presented at an A&E department or GP cooperative in the Netherlands. In usual care, medical risks and other risk factors such as environmental hazards in the home and patients' risk behaviour are not systematically registered and addressed by hospital physicians, specialists or general practitioners. Moreover, no systematic attention is currently being paid to the specific consequences of an injurious fall for the daily functioning of individual patients in their unique situation. We placed no restrictions on co-interventions.

### Effect evaluation

The primary outcome measures of the effect evaluation are number of falls and daily functioning. Number of falls is subdivided into three separate measures: the percentage of elderly people sustaining a fall during the one-year follow-up period, recurrent falls during follow-up (i.e., the percentage of elderly people sustaining two or more falls), and injurious falls during follow-up (the percentage of elderly people receiving medical care after a fall). Falls are recorded continuously by means of a fall calendar during the twelve-month follow-up period. Subjects are called monthly to report their falls as recorded on the fall calendar relating to the previous month.

We decided to measure daily functioning by means of the Frenchai Activity Index (FAI) [15], in contrast to Close et al. [5], who used the Barthel Index. Our reason for choosing this instrument was that the FAI has proved to be suitable for the general population of elderly people [14] and has at least two advantages over the Barthel Index. One is that the Barthel Index shows a ceiling effect when applied to elderly people who have sustained a fall [5]. The other is that most activities of daily living (ADL) scales, like the Barthel Index, do not refer to complex activities like housekeeping, recreation, hobbies and social interaction. These so-called instrumental abilities (IADL) may affect the quality of life considerably, and the FAI focuses primarily on these IADL abilities [15]. The FAI is measured by means of self-administered questionnaires at baseline and after four and twelve months.

Our secondary outcome measures are: recuperation from the fall, health complaints, perceived health measured by means of the first two items of the RAND-36 [16], ADL and IADL disability measured by means of the GARS (Groningen Activity Restriction Scale) [17], mental health measured by means of the HADS (Hospital Anxiety and Depression Scale) [18,19] and quality of life measured by means of the European Quality of Life instrument (EuroQol) [20]. The secondary outcome measures are assessed by means of self-administered questionnaires at four and twelve months.

Besides the primary and secondary outcome measures, we assess some background variables which are considered to be predictors, confounders or effect modifiers. The following personal characteristics are assessed: age, sex, marital status, living alone and socio-economic status. In addition, we assess the circumstances and causes of the falls reported at the GP cooperative and/or A&E department, the consequences of the falls (using the Falls Handicap Inventory [21]), the type of injury, falls in the previous year (retrospective), the patient's height, weight, use of medication and social contacts (using an adjusted version of items 4 and 5 of the Rand Social Health Battery)[22,23], and the occurrence of life events. All background variables are measured at baseline.

### Economic evaluation

The economic evaluation in our study is being performed from a societal perspective, which implies that all costs and outcomes are taken into account if possible. The economic evaluation will be a combination of a cost-effectiveness and a cost-utility analysis. The primary outcome measure for the cost-effectiveness analysis will be the percentage of people sustaining a fall during one year of follow-up. As mentioned above, falls are recorded by means of a calendar. Within the cost-utility analysis, the effects are measured in terms of generic health-related quality of life descriptions, measured according to the standard Dutch version of the EuroQol (EQ 5-D) [20] in self-administered questionnaires at baseline and after four and twelve months. A direct value for every state of health is generated using the social tariff [24], which involves an algorithm for interpolating EuroQol results to population utilities.

We will assess programme costs, healthcare costs and patient and family costs. All costs are measured by means of a cost diary [25], in which patients continuously record volumes of health care utilisation during the twelve-month follow-up period. Subjects are asked to report their cost diary relating to the previous month during the same monthly telephone interview in which they report falls from the calendar. The volume of each category we measure will be multiplied by the cost price of each category. Cost prices are presented in Euros. Health care costs are estimated according to the Dutch guideline for cost analysis in health care research [26]. Where such guidelines are not available for a specific category, real costs or tariffs are used to estimate costs.

### Process evaluation

The process evaluation involves assessing the extent to which the intervention programme is performed according to protocol, the nature of the recommendations made to the participants, participants' compliance with these recommendations and the opinions of participants, physicians and therapists about the intervention programme and recommendations. Data on these topics are collected using the following methods: structured registration forms for the medical and occupational parts of the intervention programme; self-administered evaluation forms filled in by the participants after the medical intervention; interviews by telephone with the participants six weeks or longer after the recommendations are sent and interviews with all participating physicians and therapists at the end of the intervention period.

### Analysis

Data will be primarily analysed according to the intention-to-treat principle, i.e., including all participants with valid data, regardless of whether they received or did not receive the intervention. Subsequently, the results of the intention-to-treat analysis will be compared with the results of an on-treatment analysis, to assess whether protocol deviations have caused bias. Participants with documented deviations from the study protocol (i.e., participants in the intervention group who did not receive the entire intervention or participants in either the intervention or the control group with incomplete follow-up data) will be excluded from this on-treatment analysis.

Comparability between the intervention and control groups will be assessed at baseline to check for differences between the two groups. Outcome at four and twelve months will be compared between the intervention and control groups by both univariate and multivariate techniques. We will use multivariate analysis to adjust for possible differences in baseline scores and background variables between the intervention and control groups. Dropouts and losses-to-follow up will be described.

The economic evaluation will involve calculating cost-effectiveness and cost-utility ratios. The additional costs and additional benefits of the intervention programme compared with usual care will be examined by calculating incremental cost-effectiveness and cost-utility ratios. These incremental ratios represent the difference in mean costs between the intervention and usual care groups in the numerator and the difference in mean effects in the denominator[27].

Since the recruitment period is only 14 months, and the follow-up period is also relatively short (12 months), it is unlikely that there will be substantial differences between costs made by and for patients who started in the first part of the recruitment period and those who started in the last part. Therefore, discounting of costs is not required. Finally, a sensitivity analysis will be done to assess the generalisability of the assumptions made in the costing process. This sensitivity analysis, which involves calculating the upper and lower limits of the confidence interval of cost and effect variables, will allow us to explore and quantify the uncertainty not related to sampling variations.

The process evaluation will mainly be analysed by means of descriptive techniques.

### Progress of the study

Recruitment of eligible subjects commenced in December 2002 and ended in February 2004, resulting in a total of 333 eligible subjects being included in the trial. Randomisation resulted in the allocation of 166 participants to the intervention group and 167 to the control group. Of the 333 persons recruited, 105 (32 %) are male and 228 (68 %) are female.

The follow-up period is to end in May 2005. Results on the effects of the programme will be available in 2006 and will be published in the relevant journals.

## Discussion

Although the intervention has been subject of earlier research, this study will provide new information about the effectiveness in the Dutch situation. Furthermore, the results of the economic evaluation can provide information about the cost-effectiveness of the intervention and the effects on quality of life. In case of shown effectiveness and cost-effectiveness, we aim to implement this intervention into usual healthcare.

## Competing interests

The author(s) declare that they have no competing interests.

## Authors' contributions

All author's read and approved the final version of the manuscript.

MH is the investigator and performed most of the writing of this manuscript.

JH supervises the planning of the project and wrote the study design.

JD supervises the planning and methodological aspects of the project.

SA supervises the economic evaluation.

HC supervises the clinical part of the project.

JE is the principal investigator of the study.

## Pre-publication history

The pre-publication history for this paper can be accessed here:


